# Neutrophil‐to‐Lymphocyte Ratio in Bronchiectasis and Its Association With Disease Severity—A Single‐Center Retrospective Study

**DOI:** 10.1155/pm/9920691

**Published:** 2026-08-20

**Authors:** Irfan Shafiq, Ali Saeed Wahla, Mateen Haider Uzbeck, Zaid Zoumot, Mohamed Abuzakouk, Shuayb Elkhalifa, Jahnavi Bodi, Said Isse

**Affiliations:** ^1^ Respiratory Institute, Cleveland Clinic Abu Dhabi, Abu Dhabi, UAE, clevelandclinicabudhabi.ae; ^2^ Division of Infection, Immunity and Respiratory Medicine, University of Manchester, Manchester, England, UK, manchester.ac.uk

**Keywords:** bronchiectasis, disease severity, lung function, neutrophil-to-lymphocyte ratio, systemic inflammation

## Abstract

**Background:**

The neutrophil‐to‐lymphocyte ratio (NLR) is a marker of systemic inflammation and has shown promise in assessing disease severity in chronic respiratory conditions including bronchiectasis. This study explores the association between NLR and clinical markers of bronchiectasis severity, including lung function and the Bronchiectasis Severity Index (BSI).

**Objective:**

To assess the association between NLR, lung function, and bronchiectasis severity in adults with noncystic fibrosis bronchiectasis.

**Methods:**

We performed a retrospective single‐center study of adults with HRCT‐confirmed bronchiectasis identified from electronic health records. Demographic, spirometric, microbiological, BSI, and blood count data were collected. NLR was calculated from the first available complete blood count obtained during clinical stability. Correlation analyses were performed in an outlier‐truncated cohort, whereas multivariate general linear models and ordinal regression assessed independent associations with lung function and BSI severity categories.

**Results:**

The cohort included 213 patients; 99 were male and 114 female, with a mean age of 53.9 ± 20.4 years. Most patients had severe bronchiectasis by BSI category. Chronic *Pseudomonas aeruginosa* colonization was present in 33.8% and was associated with lower FEV₁% predicted. Median NLR was 2.89. NLR showed significant negative correlations with FEV₁% predicted, FVC% predicted, and FEV₁/FVC ratio, but not with continuous BSI score. After adjustment for chronic *Pseudomonas* colonization, logNLR remained independently associated with FEV₁% predicted and FVC% predicted, but not with BSI score. In ordinal regression, NLR was not independently associated with BSI severity categories. Absolute eosinophil count was the only independent predictor of advanced BSI category.

**Conclusion:**

NLR was associated with impaired lung function, but not independently with BSI. NLR may reflect physiological impairment but does not replace established severity tools such as BSI.

## 1. Introduction

Bronchiectasis is a chronic respiratory condition characterized by irreversible bronchial dilation, persistent airway inflammation, and recurrent infections. Its global prevalence has been increasing, particularly among older adults and women. According to a recent meta‐analysis, the pooled prevalence of bronchiectasis in adults is approximately 680 per 100,000 individuals **[** **1** **]**.

The clinical course of bronchiectasis is variable, with patients experiencing different degrees of symptom severity, frequency of exacerbations, and disease progression. To aid in prognostication and management, multidimensional scoring systems such as the Bronchiectasis Severity Index (BSI) and the FACED score have been developed [2, 3]. These tools incorporate clinical, radiological, and microbiological parameters to stratify patients based on disease severity and predict outcomes like mortality and hospitalization. However, their application in routine clinical practice can be limited due to the need for comprehensive data collection and complex calculations [4].

There remains a need for a simple, cost‐effective biomarker that can reliably reflect disease severity and assist in prognostication. The neutrophil‐to‐lymphocyte ratio (NLR), derived from routine complete blood counts, has emerged as a potential candidate. NLR serves as an indicator of systemic inflammation and has been associated with disease severity and outcomes in various chronic conditions, including chronic obstructive pulmonary disease (COPD), cardiovascular disease, and rheumatological diseases [5–8]. In the context of bronchiectasis, recent studies have explored the utility of NLR as a marker of disease severity. A study analyzing data from the Spanish Bronchiectasis Registry found that higher NLR values correlated with increased disease severity as measured by BSI, FACED, and E‐FACED scores. Furthermore, elevated NLR was associated with a higher frequency of exacerbations, greater colonization by pathogenic microorganisms, and poorer quality of life. These findings suggest that NLR could serve as a practical and accessible biomarker for assessing disease severity and predicting clinical outcomes in bronchiectasis patients [5].

Building upon this evidence, our study is aimed at evaluating the relationship between NLR and BSI as well as lung function parameters, specifically forced expiratory volume in 1 s (FEV1% predicted), in a cohort of patients with bronchiectasis. By investigating this association, we seek to determine the potential of NLR as a surrogate marker for pulmonary function impairment and its role in the clinical assessment of bronchiectasis severity.

## 2. Methods

### 2.1. Population

**Figure 1:**
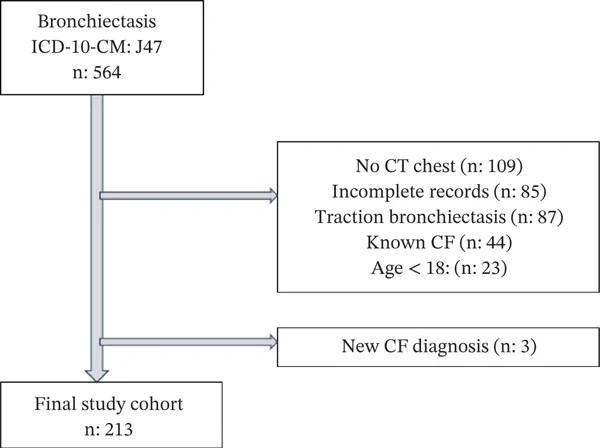
Bronchiectasis cohort flow diagram.

Electronic Health Records were queried using ICD‐10‐CM code J47, initially identifying 564 patients between April 2014 and December 2021. Following application of inclusion and exclusion criteria, 213 patients were retained for final analysis (Figure 1). Inclusion criteria comprised a radiologically confirmed diagnosis of bronchiectasis on high‐resolution computed tomography (HRCT) and a minimum of three follow‐up visits to the pulmonary clinic to ensure established longitudinal care. Exclusion criteria comprised age less than 18 years, a prior or new diagnosis of cystic fibrosis, and secondary traction bronchiectasis due to interstitial lung disease.

The study was approved by the Cleveland Clinic Abu Dhabi Research Ethics Committee (Approval Number A‐2020‐080), and all procedures were conducted in accordance with the Declaration of Helsinki. Due to the retrospective observational nature of the study, waiver of consent was applied for and approved by REC. A preliminary preprint version of this study′s methodology and initial findings has been previously deposited on Research Square [9].

### 2.2. Study Variables

Demographic data—including age, sex, and body mass index (BMI)—were collected for all participants. Clinical parameters included spirometric measures such as FEV1% predicted, forced vital capacity (FVC%), and the FEV1/FVC ratio. Microbiological data were obtained from sputum cultures, and bronchiectasis severity was assessed using the BSI. Sputum cultures were obtained from spontaneously expectorated samples collected during routine clinic visits. Colonization was defined as two or more consecutive positive sputum cultures for the same organism, or a single positive culture following a failed eradication attempt. Peripheral blood counts were reviewed to extract absolute neutrophil and lymphocyte counts. The NLR was calculated as a marker of systemic inflammation. The CBC used to derive NLR was the first available during a period of clinical stability, defined as a clinic visit at which the patient had no evidence of acute exacerbation—specifically, no clinician‐documented increase in cough frequency, sputum volume or purulence, dyspnea, or fever.

### 2.3. Statistical Methods

Continuous variables are presented as mean ± standard deviation for normally distributed parameters (such as age, BMI, and spirometric percentages) and as median and interquartile range (IQR) for highly skewed parameters, as determined by formal Shapiro–Wilk normality testing. Given the non‐normal distribution and marked variability of the raw NLR data, the variable was log‐transformed (log10NLR) to satisfy parametric assumptions for primary linear tracking. To evaluate data stability, a formal sensitivity analysis was executed for all initial linear tracking: A boxplot distribution analysis identified a tier of extreme upper anomalies (defined as raw NLR > 40); bivariate and multivariate Pearson correlation matrices were subsequently calculated utilizing this outlier‐truncated cohort to establish baseline relationships. Consequently, this outlier‐truncated sensitivity subcohort of *n* = 204 was used for primary correlation modeling, whereas the full cohort (*n* = 213) was utilized for all multivariate regression analyses.

To evaluate the independent predictive value of NLR on lung function and continuous clinical variables, a multivariate general linear model (GLM) was constructed with log10NLR as the primary covariate, adjusted for key clinical confounders including chronic *Pseudomonas aeruginosa* colonization status. To actively address and correct for significant underlying heteroscedasticity identified via Levene′s test within our interaction tracking models, the final GLM parameters were calculated utilizing Huber–White standard errors (HC3 covariance matrix estimation), ensuring the validity of our standard error limits and partial eta‐squared (*η* ^2^) effect sizes.

Finally, a multivariate ordinal regression model utilizing a standard logit link function was constructed to evaluate the independent association between clinical predictors and BSI categories (mild, moderate, and severe). This proportional odds framework simultaneously incorporated the raw NLR ratio, absolute neutrophil count, absolute lymphocyte count, absolute peripheral eosinophil count, age, BMI, and the FEV1/FVC ratio. The raw NLR ratio was deliberately utilized within this specific framework because ordinal regression models do not assume normal distribution parameters for continuous covariates, and maintaining the raw metric preserves a direct, clinically interpretable unit scale for the resulting parameters. Model assumptions were verified through goodness‐of‐fit assessments and the test of parallel lines to confirm the proportional odds criteria. Odds ratios (OR) and accompanying 95% confidence intervals (CI) were calculated and reported for all parameter estimates.

To control for inflation of the Type I error rate due to multiple testing, a hierarchical testing strategy was employed. For the primary multivariate ordinal regression framework, a Bonferroni‐adjusted alpha threshold was established for the seven independent predictors (alpha = 0.05/7 = 0.0071); *p* values below this adjusted limit were considered robust against multiple comparison inflation. Data were analyzed using IBM SPSS Statistics Version 27.0.1.0.

## 3. Results

The study cohort consisted of 213 patients with bronchiectasis, including 99 males (46.5%) and 114 females (53.5%). The mean age was 53.9 ± 20.4 years, and the average BMI was 26.4 ± 6.7 kg/m^2^. Lung function testing showed a mean FEV1% predicted of 62.4 ± 22.6, an FVC% predicted of 66.7 ± 19.7, and a mean FEV1/FVC ratio of 76.7 ± 17.7, with mild sex differences noted (Table 1).

**Table 1:** Baseline characteristics of study participants (*N* = 213).

Characteristic	Overall (*n* = 213)	Male (*n* = 99)	Female (*n* = 114)
Age (years), mean ± SD	53.9 ± 20.4	54.2 ± 19.8	53.7 ± 21.0
BMI (kg/m^2^), mean ± SD	26.4 ± 6.7	25.8 ± 5.9	26.9 ± 7.3
Lung function			
FEV1% predicted, mean ± SD	62.4 ± 22.6	60.1 ± 23.2	64.3 ± 22.0
FVC% predicted, mean ± SD	66.7 ± 19.7	64.5 ± 20.1	68.6 ± 19.2
FEV1/FVC ratio, mean ± SD	76.7 ± 17.7	74.9 ± 18.3	78.2 ± 17.0

**Figure 2:**
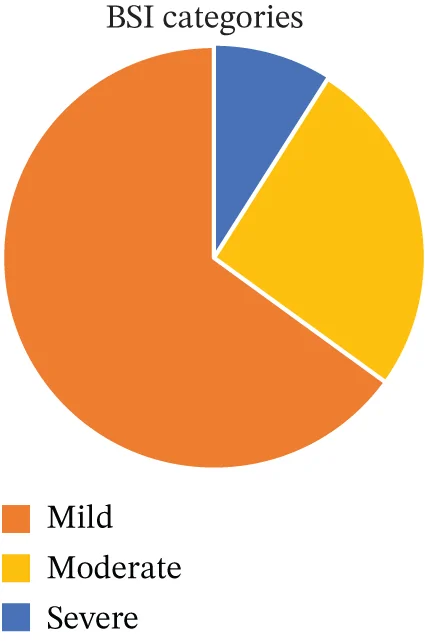
Bronchiectasis Severity Index (BSI) distribution.

**Figure 3:**
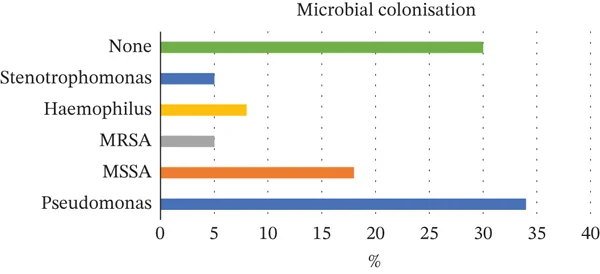
Microbiological isolates in study population.

BSI categorized 19 patients (8.9%) as having mild disease, 56 patients (26.3%) as moderate, and 138 patients (64.8%) as severe (Figure 2). Sputum culture demonstrated colonization with *Pseudomonas aeruginosa* as the most common isolate, found in 33.8% of patients. Methicillin‐sensitive *Staphylococcus aureus* (MSSA) was identified in 18.3%, methicillin‐resistant *Staphylococcus aureus* (MRSA) in 4.7%, *Haemophilus influenzae* in 8.0%, and *Stenotrophomonas maltophilia* in 5.2% (Figure 3). Patients with chronic *Pseudomonas aeruginosa* airway colonization had significantly lower mean baseline lung function compared to their noncolonized counterparts (FEV₁% predicted 54.2 ± 21.1 vs. 66.4 ± 22.4, *p* < 0.01).

The median NLR across the cohort was 2.89 (IQR 5.32). Correlation analysis within the outlier‐truncated sensitivity cohort (*n* = 204) revealed that NLR demonstrated a modest yet statistically significant negative correlation with both FEV₁% predicted (*r* = −0.370, *p* < 0.001) and FVC% predicted (*r* = −0.376, *p* < 0.001) (Table 2). A significant, low‐degree negative correlation also was present between NLR and the FEV₁/FVC ratio (*r* = −0.221, *p* = 0.003), whereas no correlation was observed between NLR and continuous raw BSI scores (*r* = 0.137, *p* = 0.051). Additionally, a low‐degree but statistically significant positive correlation was also demonstrated between NLR and absolute peripheral eosinophil counts (*r* = 0.154, *p* = 0.028).

**Table 2:** Correlation analysis between NLR and clinical parameters.

Variable intersected with NLR ratio	Pearson′s *r*	*p* value
FEV1% predicted	−0.370	< 0.001
FVC% predicted	−0.376	< 0.001
FEV1/FVC ratio	−0.221	0.003
BSI score	0.137	0.051
Eosinophil count	0.154	0.028

Multivariate analysis adjusting for chronic *Pseudomonas aeruginosa* colonization confirmed that logNLR was a significant independent predictor of FEV₁% predicted (*F* = 10.585, *p* = 0.001, partial *η* ^2^ = 0.057) and FVC% predicted (*F* = 13.293, *p* < 0.001, partial *η* ^2^ = 0.071). Following adjustment for microbiological status, the independent linear associations between logNLR and the FEV₁/FVC ratio (*F* = 3.327, *p* = 0.070), absolute eosinophil count (*F* = 2.617, *p* = 0.108), and continuous raw BSI scores (*F* = 0.676, *p* = 0.412) did not reach statistical significance (Table 3).

**Table 3:** Multivariate analysis of log_NLR associations.

Dependent variable	*F* value	*p* value	Partial *η* ^2^
FEV1% predicted	10.585	0.001	0.057
FVC% predicted	13.293	< 0.001	0.071
FEV1/FVC ratio	3.327	0.070	0.019
Eosinophil count	2.617	0.108	0.015
BSI score	0.676	0.412	0.004

The etiology × logNLR interaction term was nonsignificant (*F* = 1.34, *p* = 0.215), indicating no evidence that the association between logNLR and BSI scores varied across different underlying disease etiologies. While the preliminary model explained minimal baseline variance (adjusted *R* ^2^ = 0.05) and displayed significant heteroscedasticity via Levene′s test (*p* = 0.003), the final parameters were recalculated utilizing Huber–White standard errors (HC3). This statistical correction accounts for unequal error variances, ensuring the stability and validity of the nonsignificant interaction despite the underlying heteroscedasticity.

The multivariate ordinal regression model demonstrated an excellent fit to the data (*p* = 0.004) and successfully satisfied the proportional odds assumption (*p* = 0.252). Within this adjusted framework, the raw NLR ratio was not independently associated with bronchiectasis severity categories (*p* = 0.672, OR = 0.98, 95% CI 0.91–1.07). Standalone absolute neutrophil count (*p* = 0.260), lymphocyte count (*p* = 0.480), age (*p* = 0.481), BMI (*p* = 0.159), and the FEV₁/FVC ratio (*p* = 0.648) also showed no independent association with severity tiers. Instead, absolute peripheral eosinophil count was the only independent predictor of advanced BSI categories (*p* = 0.002, OR = 1.002, 95% CI 1.001–1.003) (Table 4).

**Table 4:** Ordinal regression analysis of predictors for bronchiectasis severity (BSI categories).

Variable	Estimate (SE)	Wald Chi‐square	*p*value	Odds ratio (95% CI)
NLR ratio	−0.018 (0.041)	0.179	0.672	0.98 (0.91–1.07)
Eosinophils	0.002 (0.001)	9.987	0.002∗	1.002 (1.001–1.003)
Neutrophils	0.068 (0.060)	1.268	0.260	1.07 (0.95–1.19)
Lymphocytes	0.098 (0.138)	0.499	0.480	1.10 (0.84–1.45)
Age	0.005 (0.008)	0.497	0.481	1.01 (0.99–1.02)
BMI	−0.033 (0.024)	1.980	0.159	0.97 (0.92–1.01)
FEV1/FVC ratio	−0.004 (0.008)	0.208	0.648	1.00 (0.98–1.01)

*Note:* Model characteristics: link function: logit. Model fit Chi − square = 20.965 (*p* = 0.004); goodness‐of‐fit Pearson *p* = 0.471; Nagelkerke pseudo *R* ^2^ = 0.125. Proportional odds assumption upheld (parallel lines test *p* = 0.252).

## 4. Discussion

Our study explored the relationship between the NLR and clinical indicators of disease severity in adult patients with bronchiectasis. The microbiological data indicated a predominance of *Pseudomonas aeruginosa* colonization, and the BSI classification revealed a substantial proportion of participants in the severe category, probably because our hospital is the main referral tertiary center in UAE.

Our study demonstrates that NLR, a readily accessible marker of systemic inflammation, is significantly associated with impaired lung function in patients with bronchiectasis, as reflected by its inverse correlations with FEV1% predicted. These findings underscore the pivotal role of neutrophilic inflammation in bronchiectasis pathogenesis, a phenomenon well‐documented in prior studies [10]. Neutrophils drive airway damage through the release of proteases and reactive oxygen species, leading to progressive bronchial dilation and parenchymal destruction [10]. The strength of NLR′s association with lung function in our cohort suggests its potential utility as a surrogate marker for tracking disease progression, particularly in settings where frequent spirometry is impractical. However, the absence of a significant correlation between NLR and the BSI score highlights the limitations of relying solely on inflammatory biomarkers to capture the multifactorial nature of bronchiectasis severity [2].

We used multivariate linear regression with NLR value as an independent variable to assess the dependence of various clinical variables including BSI and the spirometric parameters on it. The results showed an inverse relationship between the NLR and the FEV1% predicted but failed to show a significant link between the NLR and BSI. NLR′s relationship with BSI did not vary significantly by etiology. However, low explanatory power (*R* ^2^ = 0.06) and heteroscedasticity limit conclusions. One problem with such analysis is that it treats BSI score as a continuous variable. However, since there is no established minimal clinically important difference (MCID) for BSI, it is hard to say what small changes in BSI mean on a clinical level [2]. As we know that the grouping of BSI scores has definite clinical implications for patient care and prognosis, we utilized the ordinal regression used to see if BSI′s validated categories (mild/moderate/severe) have a better causal relationship with the NLR [3]. While the crude bivariate analysis suggested a slight categorical trend, the adjusted multivariate ordinal regression model showed that standalone NLR did not demonstrate a predictive relationship with BSI categories. Instead, absolute peripheral eosinophil count emerged as the only independent predictor of advanced clinical severity tiers.

A high NLR appears to track with severity in simple models but loses its independent predictive value when its individual cell counts are included. Previously, Kwok et al. found that elevated baseline NLR predicted a higher risk of hospitalization for exacerbations over a 4‐year follow‐up in a large Asian cohort [11]. Other researchers have reported NLR levels to be higher during exacerbations and correlated with positive sputum cultures, which is understandable as acute disease exacerbations are predominantly due to neutrophilic inflammation [12]. Similarly, a large study by Martínez‐García found that higher baseline NLR was linked to more severe disease, frequent flare‐ups, and a poorer quality of life [5]. However, not all researchers have found strong correlations between NLR and disease severity scores. Coban and Gungen reported that while NLR correlated with systemic inflammation markers, it was not independently associated with FACED or BSI scores among stable bronchiectasis patients [13].

Although a statistically significant positive correlation was observed between baseline NLR and absolute peripheral eosinophil counts, the low magnitude of this linear relationship (*r* = 0.154) suggests a negligible direct clinical significance. However, our adjusted multivariate model reveals a clear clinical distinction: Standalone NLR does not have an independent relationship with disease severity, whereas absolute peripheral eosinophil count is a strong, independent predictor of BSI categories (*p* = 0.002). While several previous studies have linked eosinophilic bronchiectasis subtypes to milder disease profiles [14, 15], our findings align more closely with our previously published work where peripheral eosinophilia tracked with elevated BSI scores [16]. This regional variation is likely driven by a high baseline prevalence of atopy, concurrent asthma, and allergic bronchopulmonary aspergillosis (ABPA) phenotypes concentrated within our tertiary referral population.

The study had several limitations, including its retrospective, single‐center design, which introduces the potential for selection and information bias. The predominance of severe BSI categories likely reflects referral bias inherent to our tertiary center which is the main national referral center for complex bronchiectasis patients. Furthermore, systemic inflammation markers like NLR can be affected by various comorbidities or subclinical infections that were not adequately controlled for in this study. Finally, temporal changes in NLR and exacerbation episodes were not assessed.

Our retrospective, single‐center observational study looks at the clinical significance and limitations of the NLR in individuals with non‐CF bronchiectasis. While a modest inverse relationship exists between baseline NLR and objective spirometric measures (FEV₁% and FVC% predicted), the ordinal regression did not show any association between raw NLR and categorical BSI severity tiers. Therefore, NLR cannot replace multidimensional risk scores such as BSI at present.

**Nomenclature**
MSSA: methicillin‐sensitive *Staphylococcus aureus*
MRSA: methicillin‐resistant *Staphylococcus aureus*
Partial *η* ^2^
: effect size (0.01 = small, 0.06 = medium, and 0.14 = large)

## Author Contributions

I.S.: conceptualization, methodology, project administration, writing – original draft, supervision. A.S.W.: data curation, investigation, writing – review and editing. M.H.U.: data curation, investigation, writing – review and editing. Z.Z.: formal analysis, validation, writing – review and editing. M.A.: formal analysis, validation, writing – review and editing. S.E.: methodology, validation, writing – review and editing. J.B.: data curation, investigation. S.I.: data curation, investigation.

## Funding

No funding was received for this manuscript.

## Disclosure

A preliminary version of this manuscript has been made available as a preprint on the Research Square platform (https://www.researchsquare.com/article/rs-6691002/v1) [9].

## Conflicts of Interest

The authors declare no conflicts of interest.

## Data Availability

The data that support the findings of this study are available from the corresponding author upon reasonable request.
